# Positive allosteric GABA_A_ receptor modulation counteracts lipotoxicity-induced gene expression changes in hepatocytes *in vitro*

**DOI:** 10.3389/fphys.2023.1106075

**Published:** 2023-02-13

**Authors:** Elisabeth Rohbeck, Corinna Niersmann, Karl Köhrer, Thorsten Wachtmeister, Michael Roden, Jürgen Eckel, Tania Romacho

**Affiliations:** ^1^ German Diabetes Center, Institute for Clinical Diabetology, Leibniz Center for Diabetes Research at Heinrich-Heine-University Düsseldorf, Düsseldorf, Germany; ^2^ German Center for Diabetes Research (DZD), Partner Düsseldorf, München-Neuherberg, Germany; ^3^ CureDiab Metabolic Research GmbH, Düsseldorf, Germany; ^4^ Biological and Medical Research Centre (BMFZ), Medical Faculty, Heinrich-Heine-University Düsseldorf, Düsseldorf, Germany; ^5^ Department of Endocrinology and Diabetology, Medical Faculty and University Hospital Düsseldorf, Heinrich-Heine-University Düsseldorf, Düsseldorf, Germany; ^6^ Chronic Complications of Diabetes Lab (ChroCoDiL), Department of Nursing Sciences, Physiotherapy and Medicine, Faculty of Health Sciences, University of Almería, Almería, Spain

**Keywords:** hepatocytes, lipotoxicity, GABA_A_ receptor, NAFLD, RNA sequencing (RNAseq)

## Abstract

**Introduction:** We have previously shown that the novel positive allosteric modulator of the GABA_A_ receptor, HK4, exerts hepatoprotective effects against lipotoxicity-induced apoptosis, DNA damage, inflammation and ER stress *in vitro.* This might be mediated by downregulated phosphorylation of the transcription factors NF-κB and STAT3. The current study aimed to investigate the effect of HK4 on lipotoxicity-induced hepatocyte injury at the transcriptional level.

**Methods:** HepG2 cells were treated with palmitate (200 μM) in the presence or absence of HK4 (10 μM) for 7 h. Total RNA was isolated and the expression profiles of mRNAs were assessed. Differentially expressed genes were identified and subjected to the DAVID database and Ingenuity Pathway Analysis software for functional and pathway analysis, all under appropriate statistical testing.

**Results:** Transcriptomic analysis showed substantial modifications in gene expression in response to palmitate as lipotoxic stimulus with 1,457 differentially expressed genes affecting lipid metabolism, oxidative phosphorylation, apoptosis, oxidative and ER stress among others. HK4 preincubation resulted in the prevention of palmitate-induced dysregulation by restoring initial gene expression pattern of untreated hepatocytes comprising 456 genes. Out of the 456 genes, 342 genes were upregulated and 114 downregulated by HK4. Enriched pathways analysis of those genes by Ingenuity Pathway Analysis, pointed towards oxidative phosphorylation, mitochondrial dysregulation, protein ubiquitination, apoptosis, and cell cycle regulation as affected pathways. These pathways are regulated by the key upstream regulators TP53, KDM5B, DDX5, CAB39 L and SYVN1, which orchestrate the metabolic and oxidative stress responses including modulation of DNA repair and degradation of ER stress-induced misfolded proteins in the presence or absence of HK4.

**Discussion:** We conclude that HK4 specifically targets mitochondrial respiration, protein ubiquitination, apoptosis and cell cycle. This not only helps to counteract lipotoxic hepatocellular injury through modification of gene expression, but - by targeting transcription factors responsible for DNA repair, cell cycle progression and ER stress - might even prevent lipotoxic mechanisms. These findings suggest that HK4 has a great potential for the treatment of non-alcoholic fatty liver disease (NAFLD).

## 1 Introduction

No specific pharmacological therapy has been approved to treat non-alcoholic fatty liver disease (NAFLD), a complex disease with many environmental, but also genetic factors contributing to its origin and progression (Byrne and Targher, 2015; Targher et al., 2021). Several previous studies targeting the GABAergic system have shown promising hepatoprotective effects against liver toxicity *in vitro* and *in vivo* (Norikura et al., 2007; Shilpa et al., 2012; Hata et al., 2019). It has been reported that GABA improved mitochondrial function in parallel to an attenuation of apoptotic cell death in mice with severe acute liver injury (Hata et al., 2019). Therefore, we aimed to determine if the positive allosteric modulator (PAM) of the GABA_A_ receptor, HK4, could prevent the deleterious effects of lipotoxicity in hepatocytes. In our recent publication (Rohbeck et al., 2022) we were able to confirm the presence of the GABA_A_ receptor in HepG2, as described by Minuk and colleagues (Minuk et al., 1987; Rohbeck et al., 2022). Moreover, we showed that the GABA_A_ receptor PAM, HK4, could reduce palmitate (PA)-induced DNA fragmentation, inflammation, cell death and especially apoptosis, *in vitro* (Rohbeck et al., 2022). These effects were mediated by activation of the two transcription factors nuclear factor kappa-light-chain-enhancer of activated B Cells (NF-κB) and signal transducers and activators of transcription 3 (STAT3) (Rohbeck et al., 2022). Those transcription factors control the expression of a large number of downstream genes related to cell proliferation, survival, stress responses and inflammation (He and Karin, 2011). Therefore we hypothesize that besides these two transcription factors, HK4 might also counteract gene expression patterns induced by lipotoxic stimuli such as PA exposure.

The development of NAFLD in humans strongly correlates with the dysregulation of transcriptional regulators that affect lipid metabolism (CAR, ChREBP, C/EBPα, FXR, LXR, PPARα/γ/δ, SREBP1c, STAT5), inflammation (c-Jun, C/EBPβ, IRF1/3, NF-κB, RELA, SHP, STAT1/3), metabolic stress (ATF4/6, CYP2E1, eIF2α, IRE1α, Nrf2, Xbp1), and fibrosis (AEBP1, RUNX2, Smad/TGFβ, YAP) (Steensels et al., 2020). Besides the aforementioned transcription factors, there are plenty of genes whose expression correlates positively or negatively with aberrant pathways involved in NAFLD (Greco et al., 2008; Jonas and Schürmann, 2021). Therefore we aimed to identify PA-regulated genes and to further understand the underlying signalling pathways regulated by HK4 as a protection against PA-induced lipotoxicity. In fact, lipid intake enriched in PA, has frequently been associated with obesity, type 2 diabetes mellitus and NAFLD (Hernández et al., 2017). In detail, ingestion of saturated fat rapidly increases energy metabolism, hepatic lipid storage, and insulin resistance (Nowotny et al., 2013). It has been postulated that this metabolic change is accompanied by regulation of hepatic gene expression and signalling that may contribute to the development of NAFLD (Hernández et al., 2017). Thus, several studies have identified candidates that may target transcriptional networks as a response to palmitate (Das et al., 2010; Piccolis et al., 2019; Pérez-Schindler et al., 2022), while other studies identified a typical gene expression signature in NAFLD progression in humans mostly related to lipid metabolism, oxidative phosphorylation, inflammation, endoplasmic reticulum (ER) and oxidative stress and apoptosis pathways (Greco et al., 2008; Ryaboshapkina and Hammar, 2017). RNA-sequencing can further identify and unravel specific gene patterns and involved pathway regulations.

Therefore, the global aim of the present study was to further explore the effects of HK4 on palmitate-induced lipotoxicity in hepatocytes at transcriptional level. In detail, we have examined if the previously described preventive effect of HK4 over palmitate can be translated to the transcriptional level by taking well-known transcriptional features of NAFLD into account.

## 2 Materials and methods

### 2.1 Cell culture

HepG2 cells were obtained from Merck KGaA (Darmstadt, Germany; ECACC certified) and cultured according to the corresponding manufacturer’s instructions. During experiments, cells were cultured in serum-free medium. Cells were treated with 200 μM bovine serum albumin-conjugated palmitate (Cayman chemical, Ann Arbor, MI) alone or in combination with 10 μM of HK4 (Taros Chemicals, Dortmund, Germany) up to 30 min prior to palmitate exposure for 7 h.

### 2.2 RNA isolation

Total RNA of 5 independent experiments, each one comprising 3 treatments (untreated, PA, PA + HK4), from HepG2 cells were isolated with an automated RNA isolation machine Innupure C16 using an RNA Isolation Kit (Analytic Jena, Jena, Germany) following the manufacturer’s instructions.

### 2.3 3′-RNA-seq analysis

The total RNA samples used for 3′-mRNA Seq analyses were quantified by fluorometric measurement using the Qubit device and a RNA High Sensitivity assay (Thermo Fisher Scientific Inc. Massachusetts, USA) and quality measured by capillary electrophoresis using the Fragment Analyzer and the “Total RNA Standard Sensitivity Assay” (Agilent Technologies Inc. Santa Clara, USA). All samples in this study showed high quality in RNA Quality Numbers (RQN 10). 100 ng total RNA per sample were used for library preparation performed according to the manufacturer’s protocol using the QuantSeq 3′-mRNA-Seq Library Prep Kit FWD (Lexogen^®^, Vienna, Austria). Bead purified libraries were normalized and finally sequenced on the NextSeq550 system (Illumina Inc. San Diego, USA), using single-end sequencing with a read length of 76bp. The bcl2fastq2 tool was used to convert the bcl files to fastq files as well as for adapter trimming and demultiplexing.

### 2.4 Bioinformatic analysis

Expression analyses were conducted with CLC Genomics Workbench (version 22.0.1, QIAGEN, Venlo. NL). The reads of all samples were adapter trimmed and quality trimmed (using the default parameters: bases below Q13 were trimmed from the end of the reads, ambiguous nucleotides maximal 2). Mapping was done against the *Homo sapiens* (GRCh38) genome sequence. After grouping of samples according to their respective experimental condition, multi-group comparisons were made and statistically determined using the in-build algorithm. The resulting *p*-values were corrected for multiple testing by FDR and Bonferroni-correction. A *p*-value of ≤0.05 was considered significant.

Ingenuity Pathway Analysis (IPA, Qiagen) was performed to understand interaction networks within differentially expressed genes (DEGs). Absolute values of fold change (|FC|) ≥ 1.5, *p*-values ≤0.05 and the Fisher’s exact test for *p*-values have been considered for the identification of canonical pathways. The activation z-score helps to infer the activation states of implicated biological functions, while values ≥2 indicates an activation and a z-score ≤2 points towards the inhibition of potential upstream regulators or canonical pathways. If meaningful, a trend towards activation or inhibition (|z-score| ≥ 1.5) have been considered. Either networks have been designed by selecting corresponding DEGs of mitochondrial respiration or protein ubiquitination pathway or by adapting a proposal of IPA for cell death and cell cycle.

DAVID (Database for Annotation, Visualization and Integrated Discovery) Enrichment Analysis was used for the functional annotation of DEGs (2021 online version, https://david.ncifcrf.gov/) (Da Huang et al., 2009; Sherman et al., 2022). To ascertain the gene-enriched pathways and the potential Gene Ontology (GO) classification, terms comprising biological process, molecular functions, and signalling pathways concerning specifically from Kyoto Encyclopedia of Genes and Genomes (KEGG) database were used. The modified Fisher exact *p*-value (EASE score) ≤ 0.05 are considered strongly enriched.

### 2.5 Statistical analyses

Statistical analyses were performed using the GraphPad Prism software (Version 8.0.1, San Diego, USA). Two-way ANOVA (*post hoc* test: Dunnett’s) were used to assess statistical significant differences. Transcript levels (expressed as reads per kilobase of transcript per million mapped reads (RPKM)) were represented as mean values with standard error of the mean (SEM). *p*-values ≤0.05 were considered as statistically significant.

## 3 Results

### 3.1 Functional annotation of the gene expression profile of hepatocytes under lipotoxicity

Hepatocyte lipid metabolism, oxidative phosphorylation, inflammation, ER and oxidative stress as well as apoptosis pathways are altered during NAFLD progression (Byrne and Targher, 2015; Dewidar et al., 2020). In parallel, modular enrichment analysis of our lipotoxicity model showed that PA altered pathways related to lipid metabolism, cell cycle, NAFLD pathways in general, oxidative phosphorylation, apoptosis, oxidative stress response, peroxisome proliferator-activated receptors (PPAR) signalling, tumor protein p53 (TP53) and unfolded protein response (Table 1). Between 77 and 10 DEG are involved in the aforementioned pathways addressed by modular enrichment analysis with an increasing fold enrichment value.

**TABLE 1:** Modular enrichment analysis of genes differentially expressed by palmitate exposure.

Term	Count	*p*-value	Fold enrichment
Lipid metabolism	77	3.90E-03	1.40
Cell cycle	75	5.80E-04	1.50
Non-alcoholic fatty liver disease	24	2.50E-03	1.90
Oxidative phosphorylation	20	9.30E-03	1.90
Apoptosis	20	1.20E-02	1.80
Response to oxidative stress	17	6.50E-03	2.10
PPAR signalling pathway	14	5.70E-03	2.30
TP53 signalling pathway	13	1.20E-02	2.20
Response to unfolded protein	10	1.00E-02	2.70

Selected listings of modular enrichment analysis with their associated *p*-values and fold enrichment generated with DAVID, are sorted by the number of DEG, related to the pathway (Count). Gene ontology terms such as biological process, molecular functions, and KEGG, pathways of differentially expressed genes that are associated with palmitate-induced lipotoxicity. Data are generated from HepG2 cells treated with 200 µM palmitate for 7 h and compared to untreated control. PPAR, Peroxisome proliferator-activated receptors, TP53 = Tumor protein p53.

### 3.2 Treatment with palmitate and HK4 results in differentially regulated gene profiles

**FIGURE 1:**
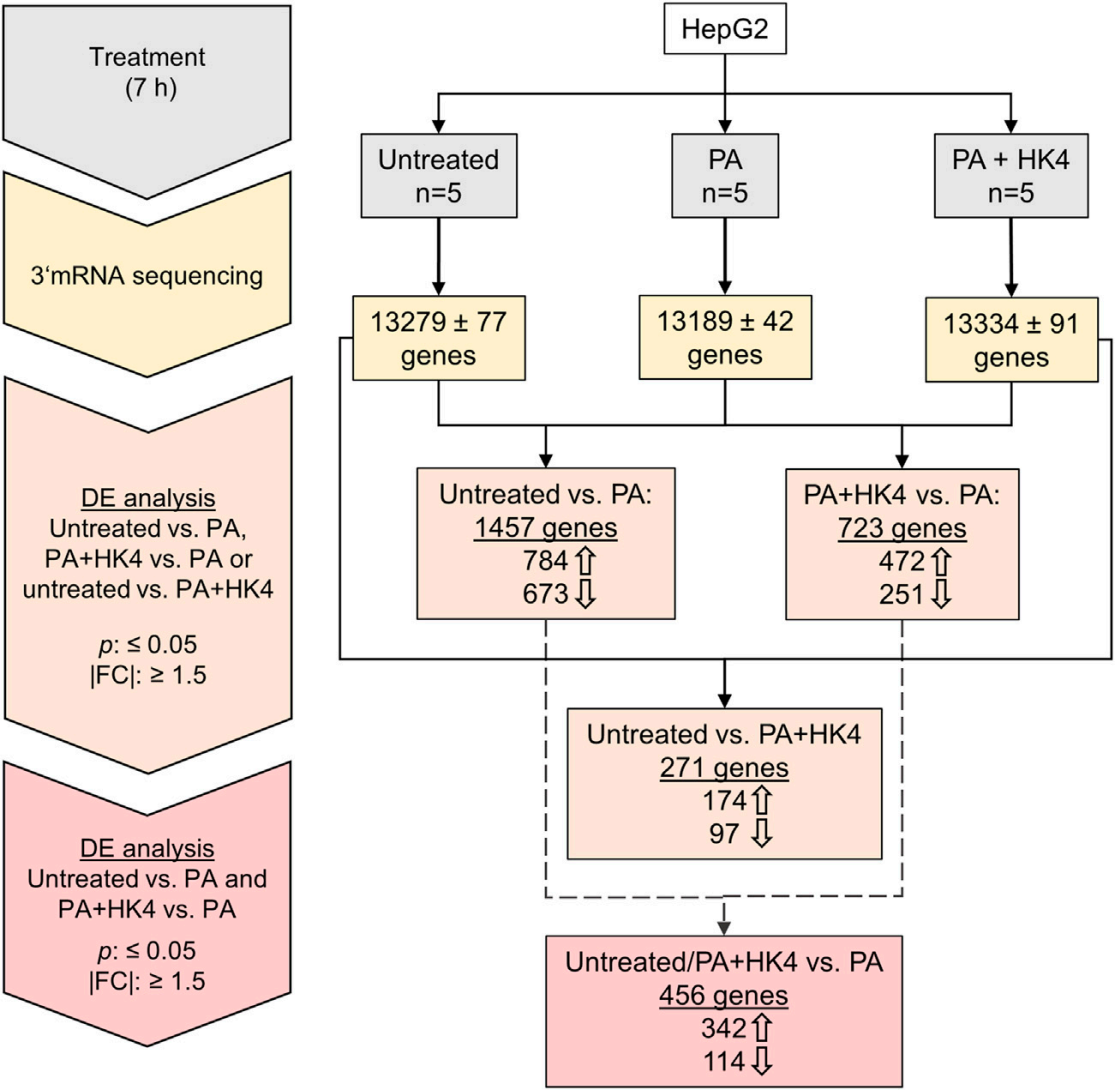
Workflow and overview of the number of genes in untreated, palmitate (PA) or PA + HK4-treated HepG2 cells. After 7 h treatment with 200 μM PA and 10 μM HK4, RNA has been isolated from five independent HepG2 cell lysates per treatment group and 3′-mRNA sequencing has been performed with Illumina NextSeq550 system. Thick arrows indicate up or downregulated genes in the first treatment group of the comparison. Differential expression (DE) of genes have been filtered by *p*-value ≤0.05 and an absolute value of fold change (|FC|) ≥ 1.5.

3′-mRNA sequencing and expression analysis identified differentially regulated protein-coding genes among untreated HepG2 cells, in PA-treated cells and PA + HK4-treated cells (Figure 1). In a differential expression analysis, genes have been filtered by a *p*-value ≤0.05 and |FC| ≥ 1.5. 1,457 genes were differentially expressed when comparing untreated and PA-treated hepatocytes, while only half of the number of genes (723) remained differentially expressed between PA alone compared to PA in combination with HK4. Interestingly, a much lower number of genes (271) are differently expressed between untreated and PA + HK4. From the 784 upregulated genes in untreated cells compared to PA-treated and the 472 upregulated genes in PA + HK4 compared to PA, 342 genes are identic. Thus, these 342 genes are downregulated when treated with PA and reversibly upregulated in the presence of HK4. In contrast, from 673 downregulated genes in untreated cells compared to PA and 251 downregulated genes in PA + HK4 compared to PA, 114 genes are identic. Thus, 114 genes are upregulated when treated with PA and reversibly downregulated in the presence of HK4. We consider those 456 genes (342 upregulated, 114 downregulated in PA + HK4) as key regulated genes by HK4 to restore initial physiological conditions from lipotoxicity.

### 3.3 Determining the transcriptome of palmitate-induced lipotoxicity and restoring of initial gene expression pattern as in untreated hepatocytes by HK4

**FIGURE 2:**
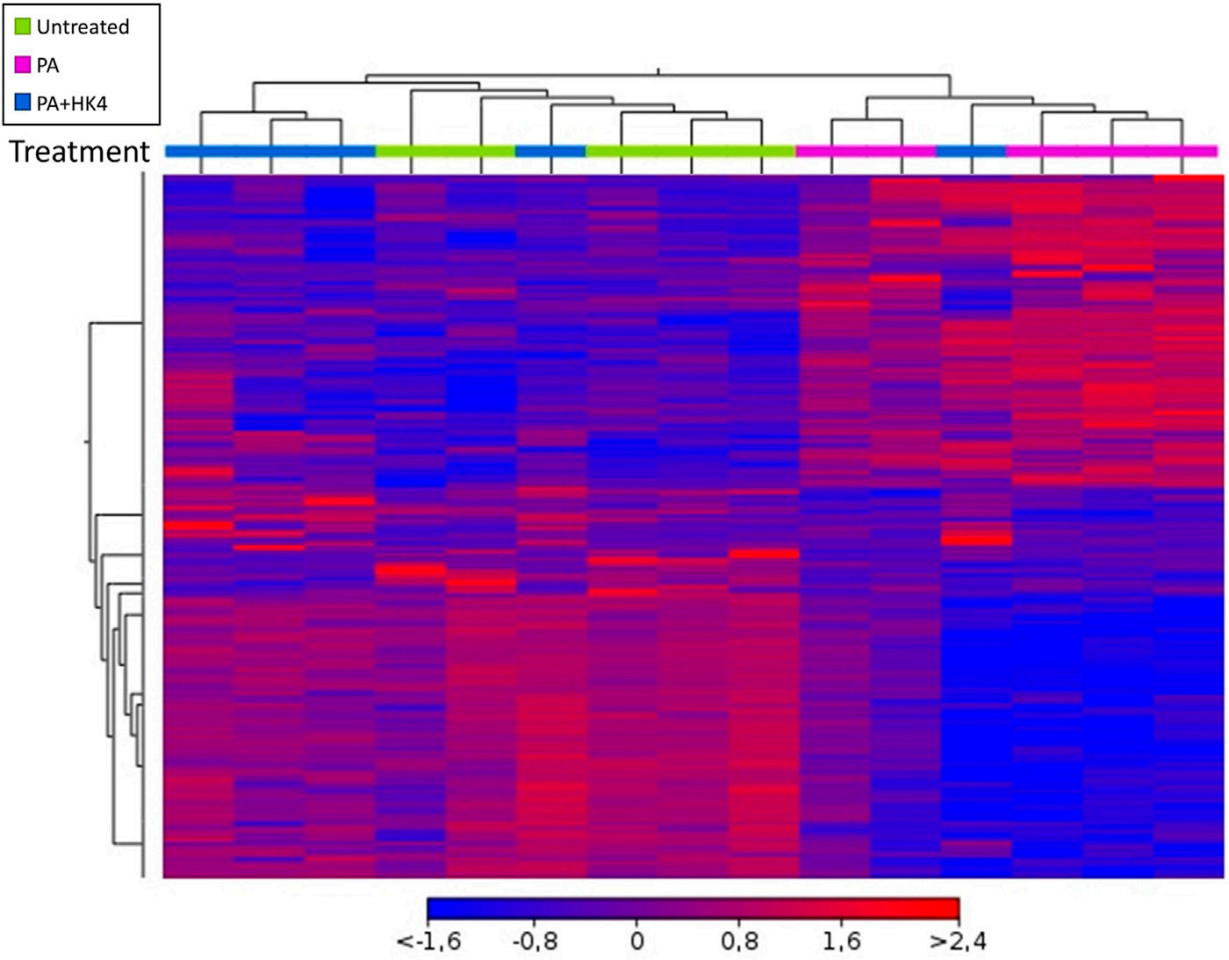
Overview of hierarchical clustering and heat map of transcription profile of HepG2 cells in response to palmitate (PA, pink) and HK4 (blue) or untreated cells (green). Euclidean metric for distance measurement was used. Changes in the abundance of 1,570 individual genes are shown, while each rectangle represents a gene. The intensity of the red and blue colors correlates with the degree of up- and downregulation, respectively. Differentially expressed genes were defined by ANOVA with a *p*-value ≤0.05 and an absolute fold change ≥1.5.

Hierarchical clustering showed distinctive gene expression profiles of untreated hepatocytes, hepatocytes exposed to PA alone or PA combined with HK4 (Figure 2). A heat map with differentially regulated genes after 7 h treatment, showed an opposite expression pattern in the majority of genes in the PA-treated group compared to untreated and PA + HK4 group. Thus, gene expression pattern of PA + HK4-treated cells is more similar to the untreated group than the PA-treated group, except for one PA + HK4 sample. In total, the heat map considered 1,570 transcripts (Supplementary Figure S1).

### 3.4 HK4-mediated modification in gene expression related to mitochondrial respiration, protein ubiquitination, cell cycle and apoptosis are crucial for protecting cells from palmitate-induced lipotoxicity

**FIGURE 3:**
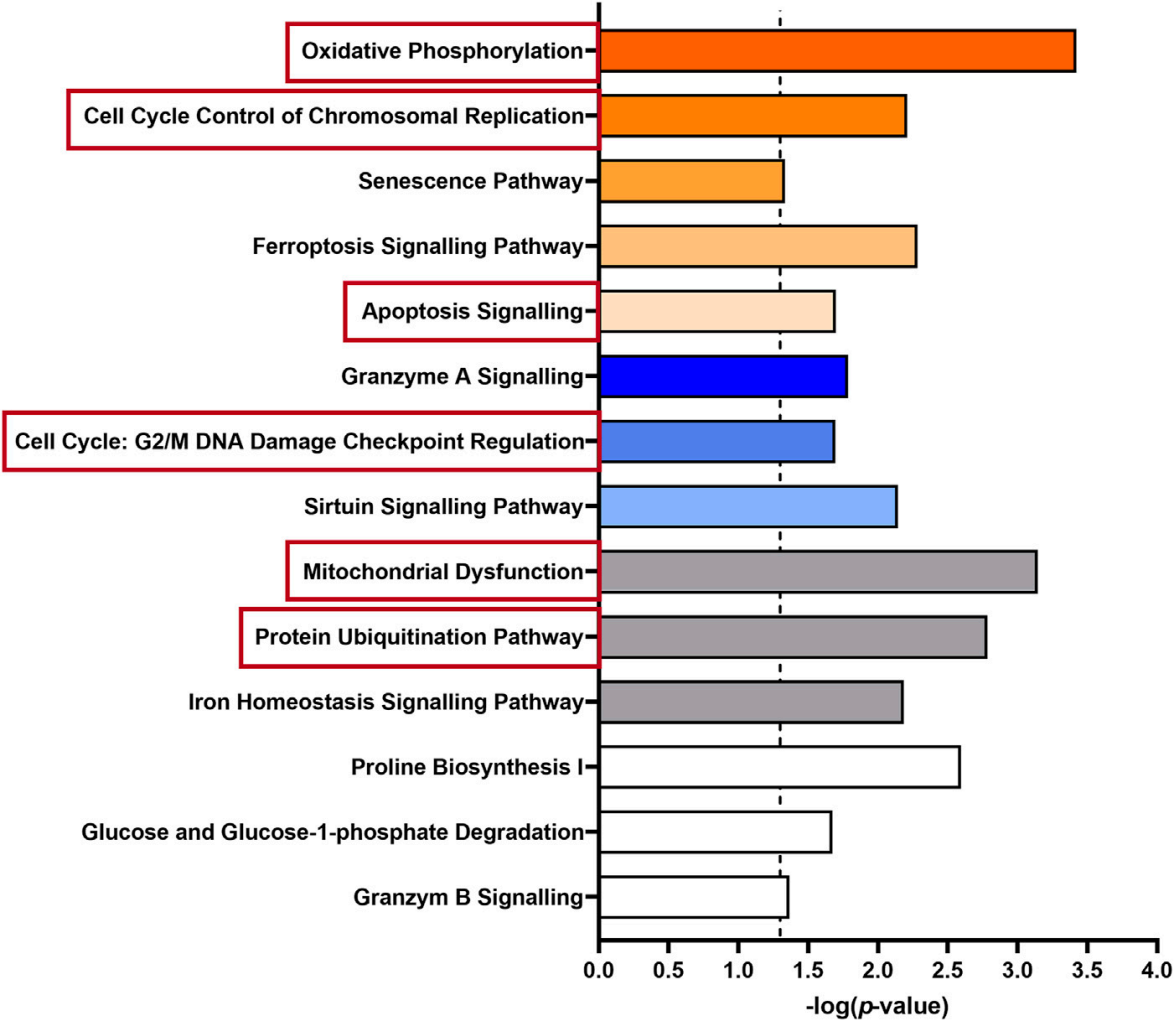
Enriched pathways of 456 differentially expressed genes in untreated or PA + HK4-treated HepG2 cells compared to PA-treated cells. The length of the bars is proportional to the significance of the association between the set of genes and the pathway, expressed by the negative logarithm of the *p*-value. Only pathways with *p* ≤ 0.05 (dotted threshold line) are shown. Upregulated pathways in untreated or PA + HK4-treated cells associated with a positive z-score are colored in orange, downregulated pathway with a negative z-score are shown in blue and pathways with no change or an unknown pattern are marked in white or grey. Selected canonical pathways which are subsequently considered in more detail are highlighted in red.

Besides the clustering of genes, we used bioinformatic approaches (IPA) to evaluate the overrepresentation of a group of genes mapping to a specific pathway compared to the total reference set of genes. Thus, we obtained an overview of the biological pathways modified by HK4 in lipotoxic conditions (Figure 3). The 456 DEGs with an up- or downregulation in PA and a reversible regulation in the presence of HK4 were analysed.

Regarding affected canonical pathways in IPA with a |z-score| ≥ 2, several DEGs are targeting and activating oxidative phosphorylation (z-score = 3, *p* = 0.0004, 9 genes), cell cycle control (z-score = 2.24, *p* = 0.006, 5 genes) or on the contrary inhibiting the caspase-independent cell death granzyme A signalling pathway (z-score = −2.24, *p* = 0.016, 5 genes) in PA + HK4 or untreated hepatocytes compared to PA-treated cells. While several other pathways related to processes such as senescence (z-score = 1.67, *p* = 0.046, 11 genes), ferroptosis (z-score = 1.13, *p* = 0.005, 8 genes), apoptosis (z-score = 0.81, *p* = 0.02, 6 genes), cell cycle regulation (z-score = −1, *p* = 0.02, 4 genes) and sirtuin signalling pathway (z-score = −0.7, *p* = 0.007, 13 genes) were significantly addressed without passing threshold |z-score| of 2. Since the underlying network also includes findings without associated directional attributes, several pathways with a considerable number of DEGs show up with unknown, unchanged or controversial pattern. Among those, mitochondrial dysfunction (*p* = 0.0007, 11 genes) protein ubiquitination (*p* = 0.002, 14 genes), iron homeostasis (*p* = 0.007, 8 genes), proline biosynthesis (*p* = 0.003, 2 genes), glucose degradation (*p* = 0.043, 2 genes) and granzyme B (*p* = 0.043, 2 genes) signalling were found.

**FIGURE 4:**
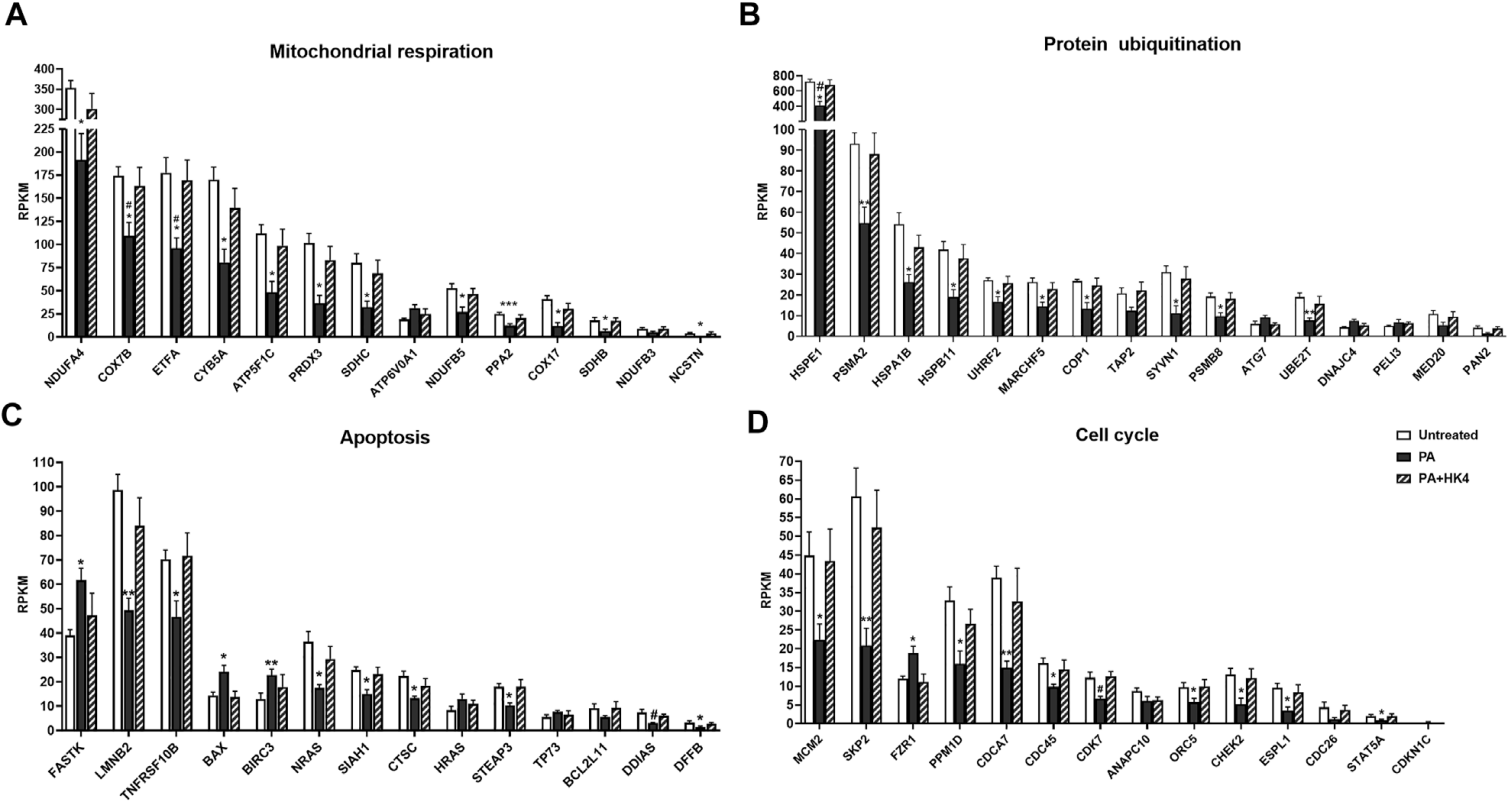
Reads per kilobase of transcript per million mapped reads (RPKM) expression values of genes related to mitochondrial respiration **(A)**, protein ubiquitination **(B)**, apoptosis **(C)** and cell cycle **(D)**. Genes were selected of the 456 DEGs among untreated (white), palmitate (PA, black) and PA + HK4 (black striped)-treated HepG2 cells and sorted by descending expression values of PA. *p*-values were calculated by two-way ANOVA; ***p ≤ 0.001, **p ≤ 0.01, *p ≤ 0.05 vs. untreated, ^#^ *p* ≤ 0.05 vs. PA + HK4-treated cells. Abbreviations of genes are explained in Supplementary Table S1.

To continue assessing the influence of HK4 on the gene expression profile, associated genes of some of the aforementioned canonical pathways were identified by IPA. Selected canonical pathways were mitochondrial respiration, protein ubiquitination, apoptosis and cell cycle related pathways (highlighted in red in Figure 3). We have taken a closer look at oxidative phosphorylation and mitochondrial dysfunction (clustered together as mitochondrial respiration), as well as protein ubiquitination, since they are the most relevant pathways according to their *p*-values and number of included genes. Additionally, we have focused on apoptosis, because we previously demonstrated the protective effect of HK4 on PA-induced apoptosis (Rohbeck et al., 2022). Since cell cycle plays a pivotal role in our lipotoxicity model with the highest number of related DEGs (Table 1) and it is closely connected to apoptosis (Caldez et al., 2020), we also took cell cycle into account. We listed genes related to the selected pathways (mitochondrial respiration, protein degradation, apoptosis, and cell cycle) suggested by IPA and DAVID together with the fold change of their expression in Supplementary Table S1. Further identified pathways were ER stress, inflammation and lipid metabolism which display few related DEGs, such as *CREB3L3*, *CHOP*, *NFKB2*, *NFKBIL1*, *IL17RC*, *ABHD3*, *PLA2G2A* (Supplementary Table S1). Out of the 456 DEGs, 14 genes (1 down-, 13 upregulated) can strongly be linked to mitochondrial respiration, 22 genes (3 down-, 19 upregulated) to protein ubiquitination pathway and 14 genes both to apoptosis (5 down-, 9 upregulated) and cell cycle (2 down-, 12 upregulated). The genes ANAPC10, BIRC3, CDC26, FZR1, SIAH1 and SKP2 are matched to several pathways of IPA (protein ubiquitination and apoptosis or cell cycle) at the same time, since the pathways strongly interact with each other. Therefore, we only depict them once for apoptosis or cell cycle, when plotting RPKM values of all associated genes in Figures 4A–D. Remarkably, in all signalling pathways considerable fewer genes are downregulated in PA + HK4-treated hepatocytes.

Most of the genes associated with mitochondrial respiration (Figure 4A) are coding components of the respiratory chain complexes like the NADH dehydrogenase (*NDUFA4*, *NDUFB3*, *NDUFB5*), the succinate dehydrogenase (*ETFA*, *SDHB*, *SDHC*), the cytochrome bc1 complex (*CYB5A*), the cytochrome c oxidase (*COX7B*, *COX17*) or the ATP synthase (*ATP5F1C*, *ATP6V0A1*). Additionally, the pyrophosphatase-coding gene *PPA2* and peroxiredoxin 3-coding gene *PRDX3* are listed as associated genes of oxidative phosphorylation and mitochondrial dysfunction. While *PPA2* regulates energy metabolism of the cell, *PRDX3* exerts an antioxidative function. From all 14 genes, only *ATP6V0A1* is downregulated in PA + HK4-treated hepatocytes compared to PA-treated cells alone.

Genes associated with protein ubiquitination (Figure 4B) depict mainly components of the E3 ubiquitin-protein ligase (*COP1*, *MARCHF5*, *PELI3, SYVN1, UHRF2*), the E2 ubiquitin-conjugated enzyme (*UBE2T*), the E1 ubiquitin-activating enzyme (*ATG7*), the proteasome (*PSMA2, PSMB8*) or as heat shock proteins (*DNAJC4, HSPA1B*, *HSPB11*, *HSPE1*). Furthermore, *TAP2* is coding a member of ATP transporter and *MED20* and *PAN2* are involved in the deubiquitination. In total, only three genes (*PELI3, ATG7* and *DNAJC4*) are downregulated in PA + HK4-treated hepatocytes compared to PA alone.

Regarding the genes associated with apoptosis (Figure 4C), 5 genes are downregulated in the presence of HK4 combined with PA compared to PA-treated cells. The most downregulated gene in this pathway, referred to its fold change (Supplementary Table S1), is the anti-apoptotic gene *BIRC3,* followed by the pro-apoptotic genes *HRAS, BAX, TP73* and *FASTK.* 9 genes (*TNFRSF10B*, *STEAP3*, *SIAH1*, *CTSC*, *BCL2L11*, *LMNB2*, *NRAS*, *DDIAS*, *DFFB*) show an upregulation when treated with PA in combination with HK4.

Regarding genes related to cell cycle (Figure 4D), two genes (*CDKN1C, FZR1*) are downregulated in untreated or PA + HK4-treated cells compared to PA-treated hepatocytes, while 11 genes are upregulated in the presence of PA + HK4. Among those, there are the cell division cycle–related genes (*CDCA7*, *CDC26* and *CDC45*), *CDK7*, *CHEK2*, *MCM2* and *ORC5*, which are mainly regulating the progression steps of the interphase, while *ANAPC10* and *ESPL1* play a role in the anaphase of the mitosis. *PPM1D* is a negative regulator of cellular stress responses.

**FIGURE 5:**
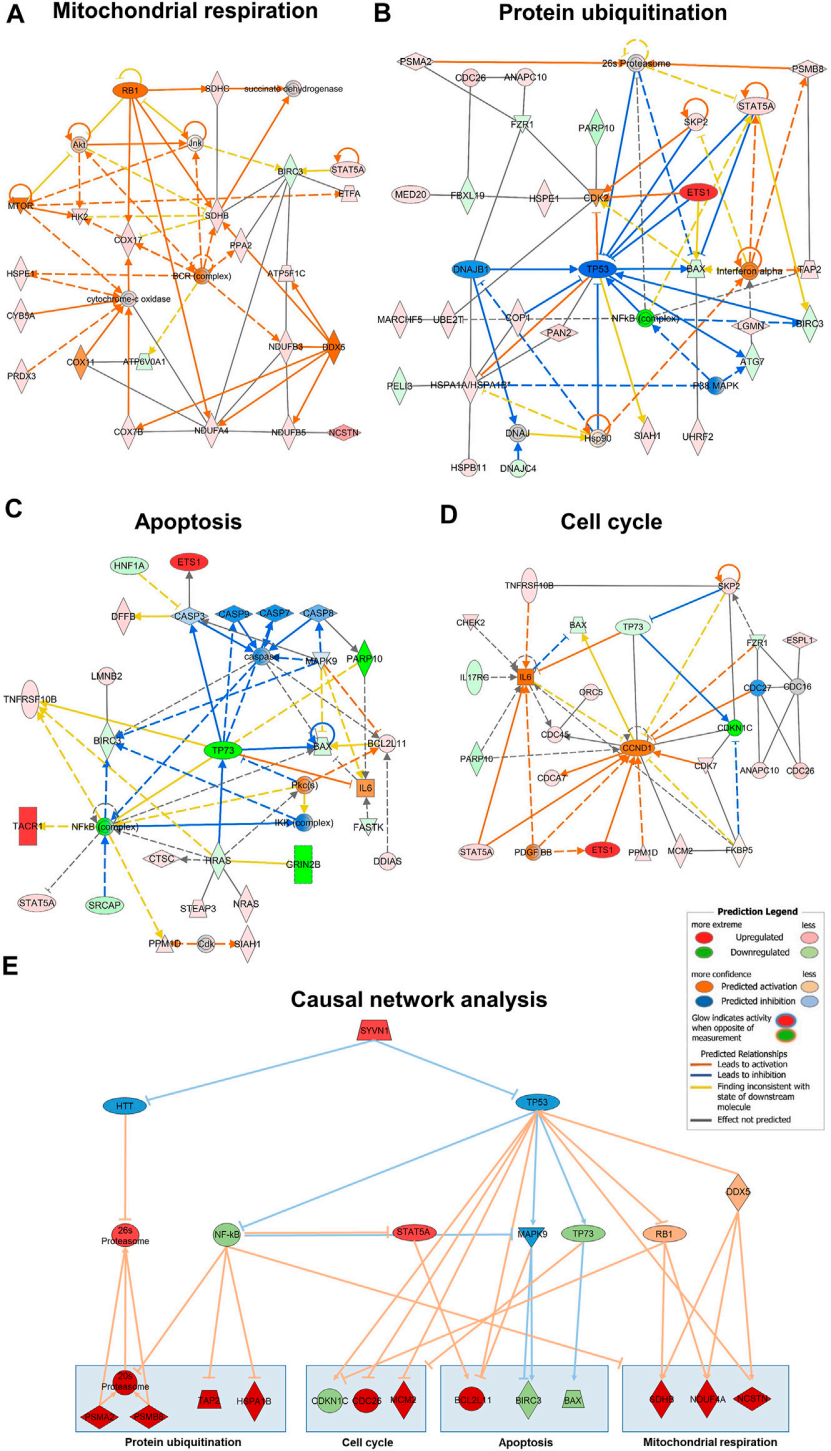
Gene interaction network map generated with IPA for mitochondrial respiration **(A)**, protein ubiquitination **(B)**, apoptosis **(C)** and cell cycle **(D)** pathways. Genes associated with one of the four pathways are represented by nodes with their shape representing the type of molecule/functional class. The causal network results of the 456 DEG identified the master regulator SYVN1 targeting the highest number of molecules in our dataset through the intermediate regulators HTT and TP53 **(E)**. Network includes up and down regulation of downstream dataset molecules. Nodes in red are upregulated in untreated/PA + HK4-treated HepG2 cells, orange nodes are predicted to be activated from Ingenuity knowledge base, green colored nodes show downregulation and blue ones are predicted to be inhibited. Orange lines between the nodes indicate an activating relationship, blue lines an inhibition. Pointed arrowheads indicate that the downstream node is expected to be activated if the upstream node connected to it is activated, while blunt arrowheads indicate that the downstream node is expected to be inhibited if the upstream node that connects to it is activated. Predicted legend is shown on the right side.

All genes of mitochondrial respiration, protein degradation, apoptosis and cell cycle have been included in networks designed with IPA with the purpose to understand how groups of data set molecules might interact (Figures 5A–D). B-cell receptor (BCR) complex, cytochrome-c oxidase, Interferon-α, IKK complex, IL 6 and CCND1, as well as the upstream regulators TP53, TP73, NF-κB, STAT5A, MAPK9, RB1 and DDX5 have been listed as additional nodes to connect the genes of the pathways.

### 3.5 Upstream regulators of PA and HK4-modified genes affect DNA repair and cell cycle

Upstream regulators cover a spectrum of molecule types (e.g., cytokines, kinases, microRNA, receptors and transcription factors) that affect the expression, transcription, or phosphorylation of other (downstream) molecules. To unravel critical mediators of lipotoxicity upstream of the 456 genes modified by PA and HK4, we identified upstream regulators with two different analyses in the IPA software. The first approach of the upstream regulator analysis (URA) determines likely upstream regulators (Table 2) that are connected to dataset genes through a set of direct or indirect relationships. URA revealed 4 potential upstream regulators of the gene expression profile by PA and HK4 with passing |z-score| threshold of 2 (Table 2). Among those potential regulators, 2 are predicted to be activated (CAB39L (z-score = 2), DDX5 (z-score = 2.65)) and 2 to be inhibited (KDM5B (z-score = −2.82), TP53 (z-score = −2,11)) with HK4 pretreatment. The most relevant upstream regulator is the tumor suppressor TP53, affecting 30 targets. The encoded protein responds to diverse ways of cellular stress to regulate expression of target genes, thereby inducing cell cycle arrest, apoptosis, senescence, DNA repair or changes in metabolism (Vogelstein et al., 2000; Marcel et al., 2010). The other transcription regulator, KDM5B, is affecting 8 target molecules from our dataset. Interestingly, KDM5B plays a role in DNA repair and the transcriptional repression of certain tumor suppressor genes (Klein et al., 2014; Li et al., 2014). DDX5, showing the highest activation z-score (2.646), is an established co-activator of TP53 and therefore having a pivotal role in orchestrating the cellular response to DNA damage and repair (Nicol et al., 2013)*.* CAB39L, the last upstream regulator of Table 2, exerts a tumor suppressive effect by inducing apoptosis and cell cycle arrest (Li et al., 2018).

**TABLE 2:** Potential upstream regulators of palmitate and HK4-induced gene expression profile.

Upstream regulator	Activation *z*-score	*p*-value	Target molecules in dataset	
			Upregulated	Downregulated
*transcription regulator*				
TP53	−2,113	4,53E-04	*BCL2L11, CDH1, CDK7, CHEK2, CITED2, CPOX, DKK1, DSN1, GLIPR1, HK2, HMGCR, HSPA1A/HSPA1B, MCM2, MPZL2, PFKFB3, PPM1D, PRNP, RAD54B, SIAH1, TAP2, TFRC, TMSB10/TMSB4X, TNFRSF10B*	*ATG7, BAX, BIRC3, ELF4, HRAS, PHLDB3, TP73*
KDM5B	−2,818	1,67E-03	*CYB5A, GCA, MCM2, MT1E, OIP5, SNRPG, SPTSSA, TMEM14A*	
*Kinase*				
CAB39L	2,000	8,59E-03	*COX17, NDUFA4, NDUFB3, NDUFB5*	
*Enzyme*				
DDX5	2,646	4,49E-05	*ATP5F1C, COX7B, FH, NDUFA4, NDUFB3, NDUFB5, SDHB*	*BAX*

Identification based on the z-score of 4 potential upstream regulators of gene expression pattern that referred to the treatment with 200 µM palmitate (PA) in combination with HK4 (10 µM) compared to PA, alone. Upstream regulators are grouped by their molecular type (including transcription regulators, a kinase and an enzyme). In practice, |z-score| ≥ 2 can be considered significant, while a negative value indicates an inactivation and a positive value an activation of the respective upstream regulator of the gene profile from PA + HK4 compared to PA, alone. TP53 = Tumor protein p53, KDM5B = lysine demethylase 5B, CAB39 L = calcium binding protein 39 like, DDX5 = DEAD-box, helicase 5.

The second approach is the causal network analysis (CNA) connecting upstream regulators to dataset molecules with the advantage of taking paths into account that involve more than one link (i.e., through intermediate regulators). Therefore, the CNA can been seen as a generalization of URA, which are used to generate a more complete picture of possible root causes for the observed expression changes (Krämer et al., 2014). In total, 19 master regulators (data not shown) are passing |z-score| threshold of 2. We plotted causal network analysis results for the “root” regulator SYVN1 which affects the most regulators (16) and target molecules (55) in our dataset (Figure 5E). SYVN1 is affecting many biological processes like response to unfolded protein, protein ubiquitination and intrinsic apoptotic signalling pathway in response to ER stress through the two intermediate regulators HTT and TP53. *HTT* (huntingtin) is known to be involved in Huntington’s disease signalling, but also required for normal development, including vesicle transport, protein trafficking, and transcriptional regulation (Rodriguez-Lebron et al., 2005). The upstream regulator TP53 not only affects 30 target molecules of our dataset in the URA, but it is also predicted to activate or inhibit 12 other upstream regulators (PRKCE, JNK, MEK, NOTCH1, NF-κB (complex), PRKAA1, GLI1, TSC2, TP73, RB1, NPM1, MAPK9). Some important activated upstream regulators by TP53, namely NF-κB, MAPK9, TP73 and RB1 are shown to interact together with STAT5A and DDX5 with selected downstream genes of mitochondrial respiration, protein ubiquitination, apoptosis and cell cycle (Figure 5E).

## 4 Discussion

PA is one of the most abundant saturated fatty acids in diet (Murru et al., 2022). Analysing the transcriptome in hepatocytes treated with PA to induce lipotoxicity, shows that short term palmitate exposure induces a specific gene expression profile. Although the effect of lipotoxicity on the induction of transcriptome responses has been analysed in hepatocytes, the impact of the novel GABA_A_ receptor PAM, HK4, on lipotoxicity has not been previously studied. Therefore, the purpose of this study was to describe gene expression changes occurring under conditions resembling lipotoxicity and to analyse the potential protection by HK4. We found that mitochondrial respiration, protein ubiquitination, apoptosis and cell cycle pathways are major targets of HK4, mainly addressed by the upstream regulators SYVN1 and TP53.

### 4.1 Differential gene expression profile of hepatocytes under PA-induced lipotoxicity and its reversion by HK4

Under conditions mimicking hepatic lipotoxicity with PA treatment, 1,457 genes were differentially expressed. In line, in HepG2 cells it has been reported that 776 genes were affected by 1 mM PA after 6 h exposure (Das et al., 2010), while others detected changes in only 11 genes after 24 h exposure of 50 μM PA (Vock et al., 2007). These differences in differential gene expression induced by palmitate are likely due concentration used, treatment duration and/or hepatocyte cell type.

A total of 456 genes were differentially expressed in PA treatment compared to untreated cells and reverted by HK4 pre-treatment. Putting these DEGs into pathophysiological context, the canonical pathways in IPA pointed towards most relevant DEGs related to mitochondrial respiration, protein ubiquitination, apoptosis and cell cycle pathways. These pathways are regulated in hepatocytes undergoing an adaptive response similarly to other studies resembling pathophysiology in NAFLD (Das et al., 2010; Mota et al., 2016; Piccolis et al., 2019).

Both oxidative phosphorylation and mitochondrial dysfunction, are listed as the top two canonical pathways of our dataset according to their *p*-value comparing PA alone and in combination with HK4.

During NAFLD electron transport chain complex expression and respiratory control ratio are decreased, while *β*-oxidation and TCA cycle activity are increased (Koliaki and Roden, 2016). This is in line with the assumption that hepatic mitochondria might upregulate their oxidative capacity at the expense of decreased coupling efficiency when transiently adapting to lipid overload (Koliaki et al., 2015; Jelenik et al., 2017). Later on, the loss of mitochondrial adaptation will favor lipid deposition and insulin resistance and in turn accelerate oxidative stress, resulting finally in non-alcoholic steatohepatitis (NASH) with impaired mitochondrial biogenesis (Fromenty and Roden, 2022). We observed that mitochondrial respiration-related genes as nicastrin (*NCSTN*), NADH dehydrogenase (*NDUFA4*) and succinate dehydrogenase (*SDHB*) were downregulated by acute palmitate exposure, exhausted from mitochondrial adaption. In mice fed with a Western style diet, mimicking NAFLD, a reduced succinate-activated respiration has been described, due to reduced *SDHB* gene expression (Staňková et al., 2021). Reduced half-life of oxidative phosphorylation subunits contributed to mitochondrial impairment in mice with NAFLD (Lee et al., 2018). It has also been described, that impaired activity of the NADH dehydrogenase, due to pathogenic mtDNA mutation, occurs in people with type 2 diabetes mellitus. (Sharma et al., 2009). However description about the gene expression of specific NADH dehydrogenase subunits (*NDUFA4*, *NDUFB3* or *NDUFB5*) in the context of NAFLD is lacking. In line with our sequencing data, NCSTN protein is overexpressed in liver in HCC leading to an enhanced cell growth and migration through Notch1 and Akt signalling pathways, however little is known about its protective effect in NAFLD (Li et al., 2020). Therefore, it can be postulated that an upregulation of the novel identified genes *NCSTN* and *NDUFA4,* as well as *SDHB* might be a protective mechanism of HK4 to restore mitochondrial capacity and boost energy supply.

The protein ubiquitination pathway is affected in our lipotoxicity model, since dysregulation of ER-protein folding and ER stress response is one of the predominant hallmarks of NAFLD progression. Specifically impaired proliferation and apoptosis in hepatocellular injury is correlated with loss of the proteasome or the inhibition of the ubiquitin-proteasome pathway (Wójcik, 2002). Thus, in the liver of people with NAFLD, inactivation of components of the ubiquitin-proteasome pathway, promotes apoptotic cell death (Joshi-Barve et al., 2003). It has also been suggested, that ATP deficiency due to reduced mitochondrial respiration contributed to inhibition of ubiquitin-proteasome and proper protein degradation and therefore activate mitophagy (Lee et al., 2018). This explains the lower expression of the protein ubiquitination-regulated genes, like *HSPA1B* and *TAP* after PA exposure and the protective effect of HK4 by elevating their expression. In line, expression of HSP70 members (like *HSPA1B*) has been proposed to take over an anti-inflammatory role in tissue-resident macrophages (like Kupffer cells) upon metabolic challenge (Brykczynska et al., 2020). There is no literature on the gene expression of TAP2 in NAFLD. However, TAP2 downregulation has been confirmed in hepatocyte-like cells from obese people, leading to abnormally metabolism and liver regeneration (Li, Y. et al., 2021).

Ji and colleagues have shown that palmitic acid could induce mitochondrial-mediated apoptosis in HepG2 by upregulating Bax and downregulating Bcl-2 (Ji et al., 2005; Panasiuk et al., 2006). Our data are in line to what was previously described, but also show that HK4 can prevent the upregulation of Bax and downregulation of Bcl-2 induced by PA. Additionally, the lower expression of *BIRC3* in HK4-treated cells might also play a protective role in our cell model, since inhibition of BIRC3 reduced hepatocellular carcinoma and progression of metastases (Fu et al., 2019; Frazzi, 2021). These findings underline the results of our recent publication where HK4 exerted an anti-apoptotic effect in HepG2 cells after 24 h of PA exposure (Rohbeck et al., 2022).

Cell division is essential for organismal growth and tissue homeostasis, especially when tissue is damaged in NAFLD (Caldez et al., 2020). Aberrations in cell cycle proteins or their regulators might even lead to HCC (Bisteau et al., 2014). In line, gene expression of *CDKN1C* in our dataset reflects protein downregulation of CDKN1C in HCC and cirrhotic liver samples (Fornari et al., 2008). Furthermore, decreased levels of *Mcm2* as a sign of dysregulated cell cycle progression has been shown in hepatocytes in NAFLD (Dabravolski et al., 2021). Inactivity or deletion of CDC26 might result in a reduced activity of anaphase-promoting complex-cyclosome, thus impairing a variety of cellular processes such as cell division, differentiation, genome stability, energy metabolism, cell death and autophagy (Zhou et al., 2016). Although less is known about the specific expression of cell cycle-related genes in patients with NAFLD, impairment (by gene mutation) and absence of cell cycle-related genes in general repress cell proliferation, which inhibits liver regeneration after acute injury or chronic damage (Caldez et al., 2020). Therefore, we can postulate that cell cycle control also plays a pivotal role in the protective effect of HK4.

### 4.2 The upstream regulators SYVN1 and TP53 control and integrate HK4 protective effects against lipotoxicity

The causal network analysis suggests that the E3 ubiquitin-protein ligase synoviolin (SYVN1, aka HDR1) is an upstream regulator. The protein encoded by this gene is involved in the ubiquitin-dependent degradation of misfolded proteins which are accumulated during ER stress and proposed as a liver metabolic regulator and a potential drug target for fatty liver disease and progressed hepatocellular carcinoma (Kikkert et al., 2004; Ji et al., 2021). In cirrhotic liver tissues upregulated SYVN1 has been implicated in the downregulation of *NRF2*, a transcription factor that combats oxidative stress (Wu et al., 2014; Bathish et al., 2022). Overexpression of SYVN1 has been proposed to ameliorate hepatic steatosis and enhanced insulin sensitivity in db/db mice (Li, K. et al., 2021). Our data suggest that upregulation of SYVN1 by HK4 pretreatment might be one mediator of the protective effect of HK4. On the contrary, other works points towards SYVN1 deletion specifically in the liver as protection against HFD-induced obesity and liver steatosis and insulin resistance in mice (Wei et al., 2018). Genome-wide mRNA sequencing revealed that SYVN1 deficiency reprograms liver metabolic gene expression profiles, including suppressing genes involved in glycogenesis and lipogenesis and upregulating genes involved in glycolysis and fatty acid oxidation (Wei et al., 2018).

Our results point towards a negative regulation of SYVN1 over HTT (Yang et al., 2007) and TP53 (Yamasaki et al., 2007). Mutant *htt* is predicted to inhibit the 26S ubiquitin proteasome system, composed of the 20S catalytic core complex and the 19S regulatory units, resulting in protein aggregation and ER stress (Valera et al., 2005; Qu et al., 2021). Thus an upregulation of the components of the proteasome complex PSMA2 and PSMB8, in the presence of HK4 might lead to proper protein degradation and avoid accumulation of misfolded proteins, which could otherwise cause hepatic steatosis (Valera et al., 2005; Rutkowski et al., 2008).

Regarding TP53, it has been described that SYVN1 sequestrates and metabolizes TP53 in the cytoplasm and negatively regulates its cellular level and biological functions, including transcription, cell cycle regulation, senescence, DNA repair and apoptosis (Yamasaki et al., 2007). As a double-edged sword TP53 takes over a dual function in both the aggravation and amelioration of NAFLD (Yan et al., 2018). Evidence also suggests that induction of TP53 improves pathophysiological conditions associated to NAFLD (Gross et al., 2017; Humpton et al., 2022). Thus, TP53 is a relevant upstream regulator in our setting orchestrating cellular signalling, like ER stress response by impaired mitochondrial function through counteraction of RB1 and NF-κB (Johnson et al., 2011). Cells lacking *Rb1* exhibit defective mitochondria and decreased oxygen consumption (Váraljai et al., 2015). Most likely, also the upstream regulator DDX5, interacting with TP53, directly regulates transcriptional activity of mitochondrial-related genes by binding at or near their promoter (Xing et al., 2020; Xu et al., 2022). Similar to our results, DDX5 expression is reported to be downregulated in palmitate-stimulated hepatocytes but also in patients with NASH (Zhang et al., 2022). Additionally, modulation of TP53-dependent pathways during prolonged metabolic stress has been also described as linked to the protein ubiquitination pathway (French and Bardag-Gorce, 2005; Lee et al., 2012). In this regard, we can speculate that through inhibition of NF-κB activation (Wadgaonkar et al., 1999), TP53 controls ubiquitination *via* the upregulation of *PSMA2, PSMB8* and *TAP2* in HK4-treated cells. Furthermore, we can postulate that TP53 might also control apoptosis either *via* interaction with TP73 and JNK pathway (MAPK9) or directly interfering with *BCL2L11* (Brockhaus and Brüne, 1999; Dhanasekaran and Reddy, 2008; Kunst et al., 2016). TP53, one of the key upstream regulators, is a well-known member of the p53 tumor suppressor family, that can inhibit cell cycle-related genes and arrest cell cycle (Kunst et al., 2016; Engeland, 2022). This might explain, why all cell cycle regulating genes except for its negative regulating inhibitor of cyclin-dependent kinases, *CDKN1C*, and *FZR1* are downregulated in lipotoxic conditions and again upregulated in the presence of HK4 in our setting (Fornari et al., 2008).

We have previously shown that the protective effects of HK4 can be mediated by downregulated phosphorylation of NF-κB and STAT3 (Rohbeck et al., 2022). Substantial evidence supports that activation of the transcription factor NF-κB and downstream inflammatory signalling pathways are involved in hepatic insulin resistance (Tilg et al., 2017). In line, upregulated NF-κB gene expression in PA-treated HepG2 cells compared to untreated (*p* = 0.004) or PA and HK4-co-treated cells (*p* = 0.089) fits with protein expression analysis of our previous study (Rohbeck et al., 2022). Also in line with our previous study, the current data indicate an involvement of STAT signaling, since STAT5A is differentially expressed in our dataset. STAT5A, likewise our recently recognized mediator of HK4 protective effect, STAT3, is known to control cell survival, differentiation, proliferation, and metabolism in response to extracellular stimuli (Wingelhofer et al., 2018). A very recent study suggests that non-canonical NF-κB activation can attenuate the hepatoprotective JAK2/STAT5 signalling (Vesting et al., 2022). We conclude from our study that mitochondrial respiration, protein ubiquitination, apoptosis and cell cycle are major targets of HK4 to minimize hepatocellular injury under a lipotoxic stimulus as PA. Our findings strongly suggest that transcription factors responsible for DNA repair and ER stress such as TP53 play a central regulatory role in hepatocyte lipotoxicity with this novel drug. Targeting those transcription factors and interfere in gene expression pattern holds great therapeutic potential for HK4 in the treatment of NAFLD.

## Data Availability

The datasets presented in this study can be found in online repositories. The names of the repository/repositories and accession number(s) can be found below: https://www.ncbi.nlm.nih.gov/, PRJNA902305.
