# Supplementary material for: Positive allosteric GABAA receptor modulation counteracts lipotoxicity-induced gene expression changes in hepatocytes in vitro
Source: Front Physiol. 2023 Feb 13;14:1106075. doi: 10.3389/fphys.2023.1106075 (PMC9968943; doi:10.3389/fphys.2023.1106075)
Supplement: Supplementary file 1 [file DataSheet2.PDF]

Gene name	HGNC object name	Identifier	Untreated vs. PA		PA+HK4 vs. PA	
			Fold change	*p* -value	Fold change	*p* -value
**Mitochondrial respiration**						
ATP5F1C	ATP synthase F1 subunit gamma	ENSG00000165629	2,17	6,46E-03	1,89	2,55E-02
ATP6V0A1	ATPase H+ transporting V0 subunit a1	ENSG00000033627	-1,81	3,61E-04	-1,58	5,69E-03
COX11	Cytochrome c oxidase copper chaperone COX11	ENSG00000166260	1,53	1,38E-02	1,43	3,64E-02
COX17	Cytochrome c oxidase copper chaperone COX17	ENSG00000138495	3,47	6,22E-04	2,64	7,73E-03
COX7B	Cytochrome c oxidase subunit 7B	ENSG00000131174	1,59	1,03E-02	1,51	2,25E-02
CYB5A	Cytochrome b5 type A	ENSG00000166347	2,04	2,07E-03	1,71	2,06E-02
ETFA	Electron transfer flavoprotein subunit alpha	ENSG00000140374	1,78	5,35E-03	1,69	1,12E-02
NCSTN	Nicastrin	ENSG00000162736	6,67	3,49E-03	6,13	5,27E-03
NDUFA4	NADH dehydrogenase (ubiquinone) 1 alpha subcomplex, 4	ENSG00000189043	1,81	6,37E-03	1,56	4,15E-02
NDUFB3	NADH dehydrogenase (ubiquinone) 1 beta subcomplex, 3	ENSG00000119013	2,08	3,36E-02	2,24	1,97E-02
NDUFB5	NADH dehydrogenase (ubiquinone) 1 beta subcomplex, 5	ENSG00000136521	1,86	9,25E-03	1,62	4,38E-02
PPA2	Inorganic pyrophosphatase 2	ENSG00000138777	2,07	6,58E-04	1,73	1,08E-02
PRDX3	Peroxiredoxin 3	ENSG00000165672	2,64	2,57E-03	2,36	7,63E-03
SDHB	Succinate dehydrogenase complex iron sulfur subunit B	ENSG00000117118	2,49	1,19E-02	2,37	1,72E-02
SDHC	Succinate dehydrogenase complex subunit C	ENSG00000143252	2,50	3,22E-03	2,17	1,28E-02
**Protein ubiquitination**						
ATG7	Ubiquitin-activating enzyme E1-like protein	ENSG00000197548	-1,60	4,44E-02	-1,71	2,18E-02
COP1	COP1 E3 ubiquitin ligase	ENSG00000143207	1,84	1,40E-02	1,64	4,69E-02
DNAJC4	DnaJ heat shock protein family (Hsp40) member C4	ENSG00000110011	-1,83	1,50E-02	-1,66	4,25E-02
HSPA1B	Heat shock protein family A (Hsp70) member 1B	ENSG00000204388	2,01	1,53E-03	1,60	3,27E-02
HSPB11	Heat shock protein family B (small) member 11	ENSG00000081870	2,11	2,19E-03	1,89	9,05E-03
HSPE1	Heat shock protein family E (Hsp10) member 1	ENSG00000115541	1,70	1,21E-02	1,59	2,97E-02
MARCHF5	Membrane associated ring-CH-type finger 5	ENSG00000198060	1,75	4,54E-03	1,50	4,00E-02
MED20	Mediator complex subunit 20	ENSG00000124641	2,05	8,84E-03	1,88	2,17E-02
PAN2	Poly(A) specific ribonuclease subunit PAN2	ENSG00000135473	2,98	1,48E-02	2,84	2,02E-02
PELI3	Pellino E3 ubiquitin protein ligase family member 3	ENSG00000174516	-1,60	3,52E-02	-1,56	4,74E-02
PSMA2	Proteasome 20S subunit alpha 2	ENSG00000106588	1,64	1,21E-02	1,55	2,78E-02
PSMB8	Proteasome 20S subunit beta 8	ENSG00000204264	1,91	1,39E-02	1,81	2,43E-02
SYVN1	Synoviolin 1	ENSG00000162298	2,66	8,02E-03	2,40	1,75E-02
TAP2	Transporter 2, ATP binding cassette subfamily B member	ENSG00000204267	1,66	4,26E-02	1,79	2,00E-02
UBE2T	Ubiquitin-conjugating enzyme E2 T	ENSG00000077152	2,24	2,73E-03	1,78	3,23E-02
UHRF2	Ubiquitin like with PHD and ring finger domains 2	ENSG00000147854	1,63	2,32E-02	1,56	3,83E-02
**Apoptosis**						
BAX	BCL2 associated X, apoptosis regulator	ENSG00000087088	-1,64	8,68E-03	-1,60	1,21E-02
BCL2L11 (BIM)	BCL2 like 11	ENSG00000153094	1,85	2,13E-02	2,03	8,00E-03
BIRC3	Baculoviral IAP repeat-containing protein 3	ENSG00000023445	-1,93	2,34E-04	-1,61	7,36E-03
CASP7	Caspase-7	ENSG00000165806	1,85	1,11E-04	1,47	1,67E-02
CASP9	Caspase-9	ENSG00000132906	3,21	3,18E-02	2,59	8,08E-02
CTSC	Cathepsin C	ENSG00000109861	1,76	4,86E-03	1,50	4,29E-02
DDIAS	DNA damage-induced apoptosis suppressor	ENSG00000165490	2,26	3,73E-03	1,84	3,07E-02
DFFB	DNA fragmentation factor subunit beta	ENSG00000169598	2,74	2,05E-02	2,59	2,96E-02
FASTK	Fas-activated serine/threonine kinase	ENSG00000164896	-1,69	1,41E-04	-1,52	2,27E-03
HRAS	HRas proto-oncogene, GTPase	ENSG00000174775	-1,77	5,69E-03	-1,60	2,24E-02
LMNB2	Lamin-B2	ENSG00000176619	1,96	1,20E-03	1,69	1,18E-02
NRAS	NRAS proto-oncogene, GTPase	ENSG00000213281	2,03	2,55E-03	1,63	3,73E-02
SIAH1	Siah E3 ubiquitin protein ligase 1	ENSG00000196470	1,70	1,64E-02	1,64	2,50E-02
STEAP3	STEAP3 metalloreductase	ENSG00000115107	1,68	2,05E-02	1,68	2,14E-02
TNFRSF10B	TNF receptor superfamily member 10b	ENSG00000120889	1,51	3,61E-02	1,58	2,06E-02
TP73	Tumor protein p73	ENSG00000078900	-1,52	3,51E-02	-1,54	3,01E-02
**Cell Cycle**						
ANAPC10	Anaphase-promoting complex subunit 10	ENSG00000164162	1,82	1,04E-03	1,61	9,88E-03
CDC26	Cell division cycle 26	ENSG00000176386	3,44	1,93E-02	2,94	4,12E-02
CDC45	Cell division cycle 45	ENSG00000093009	1,72	6,96E-03	1,63	1,59E-02
CDCA7	Cell division cycle associated 7	ENSG00000144354	2,45	5,75E-04	2,01	7,60E-03
CDK7	Cyclin dependent kinase 7	ENSG00000134058	1,71	3,56E-02	1,73	3,12E-02
CDKN1C	Cyclin-dependent kinase inhibitor 1C	ENSG00000129757	-23,50	2,35E-02	-23,25	2,43E-02
CHEK2	Checkpoint kinase 2	ENSG00000183765	2,49	3,25E-03	2,35	5,95E-03
ESPL1	Extra spindle pole bodies like 1, separase	ENSG00000135476	2,50	4,16E-03	2,18	1,50E-02
FZR1	Fizzy and cell division cycle 20 related 1	ENSG00000105325	-1,57	1,58E-02	-1,70	4,41E-03
MCM2	Minichromosome maintenance complex component 2	ENSG00000073111	1,82	1,68E-02	1,72	3,20E-02
ORC5	Origin recognition complex subunit 5	ENSG00000164815	1,66	3,94E-02	1,69	3,33E-02
PPM1D	Protein phosphatase, Mg2+/Mn2+ dependent 1D	ENSG00000170836	2,02	2,71E-03	1,66	3,05E-02
SKP2	S-phase kinase-associated protein 2	ENSG00000145604	2,72	1,94E-04	2,33	1,66E-03
STAT5A	Signal transducer and activator of transcription 5A	ENSG00000126561	2,52	1,34E-02	2,49	1,48E-02
**ER stress**						
CREB3L3	Cyclic AMP-responsive element-binding protein 3-like protein 3	ENSG00000060566	-2,65	3,89E-08	-1,50	2,04E-02
DDIT3 (CHOP)	DNA damage-inducible transcript 3 protein	ENSG00000175197	-2,06	2,08E-04	-1,43	6,64E-02
ERN1 (IRE1A)	Endoplasmic reticulum to nucleus signaling 1	ENSG00000178607	-1,89	2,84E-04	-1,37	7,42E-02
**Inflammation**						
NFKB2	Nuclear factor kappa B subunit 2	ENSG00000077150	-2,36	3,64E-03	-1,64	8,95E-02
NFKBIL1	NFKB inhibitor like 1	ENSG00000204498	-1,99	1,34E-03	-1,52	4,98E-02
IL17RC	Interleukin-17 receptor C	ENSG00000163702	-1,87	3,51E-02	-2,08	1,41E-02
IL1R2	Interleukin-1 receptor type 2	ENSG00000115590	-1,81	1,49E-02	-1,60	5,29E-02
IFNGR2	Interferon gamma receptor 2	ENSG00000159128	1,81	2,82E-02	1,58	9,21E-02
TNFRSF19	TNF receptor superfamily member 19	ENSG00000127863	1,95	1,57E-03	1,72	9,87E-03
CAPN12	Calpain-12	ENSG00000182472	3,86	2,34E-03	3,23	8,19E-03
**Lipid metabolism**						
ABHD3	Abhydrolase domain containing 3, phospholipase	ENSG00000158201	2,03	3,61E-02	2,07	3,08E-02
ACOT11	Acyl-CoA thioesterase 11	ENSG00000162390	-1,73	3,68E-02	-1,70	4,42E-02
ACOT2	Acyl-CoA thioesterase 2	ENSG00000119673	1,75	1,71E-02	1,65	3,20E-02
CHPT1	Choline phosphotransferase 1	ENSG00000111666	1,78	1,91E-02	1,70	3,04E-02
CRLS1	Cardiolipin synthase 1	ENSG00000088766	2,42	3,65E-03	1,98	2,40E-02
DGKG	Diacylglycerol kinase gamma	ENSG00000058866	-1,65	1,99E-02	-1,61	2,70E-02
MID1IP1	MID1 interacting protein 1	ENSG00000165175	1,72	1,34E-02	1,65	2,27E-02
MOGAT2	Monoacylglycerol O-acyltransferase 2	ENSG00000166391	-1,59	5,57E-03	-1,57	7,11E-03
PC	Pyruvate carboxylase	ENSG00000173599	-1,55	2,15E-02	-1,52	2,85E-02
PI4K2B	Phosphatidylinositol 4-kinase type 2 beta	ENSG00000038210	2,13	3,09E-03	2,07	4,34E-03
PISD	Phosphatidylserine decarboxylase	ENSG00000241878	1,99	1,05E-02	1,76	3,53E-02
PLA2G2A	Phospholipase A2 group IIA	ENSG00000188257	-1,59	7,40E-03	-1,54	1,35E-02
