# Supplementary material for: Positive allosteric GABAA receptor modulation counteracts lipotoxicity-induced gene expression changes in hepatocytes in vitro
Source: Front Physiol. 2023 Feb 13;14:1106075. doi: 10.3389/fphys.2023.1106075 (PMC9968943; doi:10.3389/fphys.2023.1106075)
Supplement: Supplementary file 2 [file Table1.DOCX]

Supplementary Material

**Supplementary Figure 1.** Hierarchical clustering and heat map of transcription profile of HepG2 cells in response palmitate (PA, pink) and HK4 (blue) or untreated cells (green) with 1570 individual listed genes. Euclidean metric for distance measurement was used. Changes in the abundance of genes are shown, while the intensity of the red and blue colors correlates with the degree of up- and downregulation, respectively. Differentially expressed genes were defined by ANOVA with a *p*-value ≤ 0.05 and an absolute fold change ≥ 1.5.

**Supplementary Table 1.** Differentially expressed genes of untreated, palmitate (PA) and PA+HK4-treated hepatocytes. Selected genes, differentially expressed between untreated and PA or between PA+HK4 and PA, are listed together with their object name from HUGO Gene Nomenclature Committee (HGNC) database, identifier, respective fold changes and *p*-values. Genes were classified into groups for mitochondrial respiration, protein ubiquitination, apoptosis, cell cycle, ER stress, inflammation and lipid metabolism. Except for yellow marks, genes were filtered with at least 1.5-fold change and a *p*-value ≤ 0.05.
