# Supplementary material for: Positive allosteric GABAA receptor modulation counteracts lipotoxicity-induced gene expression changes in hepatocytes in vitro
Source: Front Physiol. 2023 Feb 13;14:1106075. doi: 10.3389/fphys.2023.1106075 (PMC9968943; doi:10.3389/fphys.2023.1106075)

Supplementary Figure 1

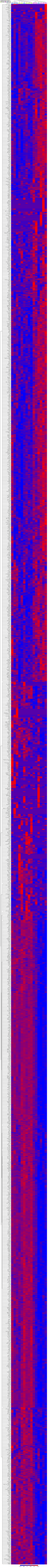

Supplement: Supplementary file 3 [file DataSheet1.PDF]
